# Sustainability of zinc coverage for acute childhood diarrhea in Bangladesh and other low- and middle-income countries: one decade following the SUZY project

**DOI:** 10.1371/journal.pgph.0004265

**Published:** 2025-02-10

**Authors:** Keith Beam, Nicole Hsu, Amandari Kanagaratnam, Charles Larson, Tracey Koehlmoos

**Affiliations:** 1 Uniformed Services University of the Health Sciences, Bethesda, Maryland, United States of America; 2 United States Air Force School of Aerospace Medicine, Fairborn, Ohio, United States of America; 3 Defense Health Agency, Public Health Directorate, Falls Church, Virginia, United States of America; 4 Henry M. Jackson Foundation for the Advancement of Military Medicine Inc., Bethesda, Maryland, United States of America; 5 McGill University, School of Population and Global Health, Montreal, Quebec, Canada; International Medical University, MALAYSIA

## Abstract

Oral zinc is a proven effective treatment for diarrheal illness, and long-term monitoring is key to evaluating the success of efforts to scale up zinc treatment. We examine zinc coverage for diarrheal illness in Bangladesh since the conclusion of the Scaling Up Zinc for Young Children (SUZY) project in 2008 and provide an overview of other countries’ zinc scale-up programs to compare the long-term effectiveness of SUZY. We used data from the Bangladesh Demographic and Health Surveys from 2005–2022 to examine the proportion of children under five receiving zinc treatment for diarrheal illness and evaluate disparities in zinc coverage by urbanicity and wealth quintile. We used a qualitative framework synthesis to compare the SUZY project with national or large-scale zinc scale-up programs in other low- and middle-income countries (Ghana, India, Kenya, Nepal, Nigeria, Uganda). This method for synthesizing qualitative and quantitative data was used to break down components of the SUZY project and other national or large-scale zinc scale-up programs. In Bangladesh, zinc coverage has continued to increase since the conclusion of the SUZY project, disparities in coverage between urban and rural areas and across wealth quintiles have been resolved, and the prevalence of diarrheal illness has decreased from 10·8% in 2007 to 4·8% in 2022. The countries with the highest zinc coverage (Bangladesh, Kenya, Uganda) had national rather than regional scale-up campaigns. Our findings demonstrate the long-term success of the SUZY project and provide insights into best practices for impactful zinc scale-up programs including significant pre-launch implementation research addressing key knowledge gaps and partnering with research organizations. Long-term monitoring of scale-up campaigns is important to determine if these interventions can become socially embedded and self-sustaining, improving health outcomes in the long run.

## Introduction

Globally, diarrheal illness remains the third leading cause of death in children under five years old; over half a million children die annually from this largely preventable cause [1]. The therapeutic effects of oral zinc for the treatment of acute and persistent diarrhea are well established. In 2004, the World Health Organization (WHO) and the United Nations International Children’s Emergency Fund (UNICEF) revised their recommendations to include zinc for the treatment of acute or chronic childhood diarrhea (20mg daily for 10-14 days for children 6 months to five years of age) [2–5]. In response, several national and international development efforts have been dedicated to scaling-up the use of zinc in combination with oral rehydration salt (ORS) for the management of diarrheal illnesses. The International Centre for Diarrhoeal Research, Bangladesh (icddr,b) launched the first national scale-up of zinc treatment in 2003. The Scaling Up Zinc for Young Children (SUZY) project had the aim of covering all children in Bangladesh from six months to under the age of five with zinc treatment for any episode of diarrheal illness [6]. The SUZY project was a unique collaborative partnership between public, private, non-governmental organizations (NGOs), and national agencies led by icddr,b, funded by the Bill and Melinda Gates Foundation, and supported by the Bangladeshi Ministry of Health and Family Welfare, Acme Laboratories, and Dhansiri Communications Ltd [7]. The SUZY program concentrated solely on increasing zinc coverage for childhood diarrhea, promoting a single innovative zinc product and using a comprehensive marketing strategy that included various media formats. Its collaboration with icddr,b facilitated extensive pre-implementation research, which informed the program’s approach and messaging.

Early success was reported, with caretaker awareness of zinc as a treatment for childhood diarrhea increasing from less than 10% at baseline in all communities to 50% in rural areas and to 90% in urban non-slum areas, at 10 months after the launch of a multifaceted mass media zinc promotion campaign [8]. By 23 months following the launch of the national scale-up campaign, 10% of rural, 20% of urban slum or smaller municipalities, and 25% of larger municipality urban non-slum children under the age of five, received zinc for the treatment of their diarrheal illness episode [8]. While the use of zinc treatment levelled off by the end of the first year in urban non-slum and smaller municipal households, a steady increase in zinc usage was observed within the most vulnerable rural and urban slum children throughout the following two year period [8]. Concurrently, a study of over 25,000 children hospitalized and treated with zinc at icddr,b’s Dhaka hospital for acute childhood diarrhea reported no serious adverse effects [9].

However, since publication of the original SUZY reports and closure of the project in 2008, limited data are available to evaluate the long-term effectiveness of this program. Long-term monitoring of scale-up campaigns such as the SUZY project is important to determine if these interventions can become socially embedded and self-sustaining, and to highlight lessons learned to inform other countries embarking on similar initiatives. This paper describes zinc coverage for diarrheal illness in Bangladesh since the conclusion of the SUZY project and provides an overview of other national zinc scale-up programs reported in the published literature.

## Methods

This was a retrospective, mixed methods evaluation of programs that were designed to scale-up zinc coverage for childhood diarrhea. The primary objective was to evaluate the impact of the SUZY project on diarrheal treatment in Bangladesh. Specifically, the proportion of children under five receiving zinc treatment for a diarrheal illness, disparities in zinc coverage, and potential decrease in use of ORS over time were assessed. The secondary objective was to identify other countries that have implemented zinc scale-up campaigns and to compare the effectiveness of these programs, using qualitative framework synthesis. This study was found exempt by the Institutional Review Board of the Uniformed Services University.

### Objective #1 - SUZY project long-term follow-up

#### Data sources and outcome variable.

Full datasets for the Bangladesh Demographic and Health Surveys (BDHS) were obtained for 2007, 2011, 2014, and 2017–18 in addition to the preliminary report for the 2022 BDHS to conduct a cross-sectional ecologic study evaluating the long-term impact of the SUZY project [10–14]. DHS data is deidentified and aggregated for secondary analysis so did not require special handling. This data was combined into a single dataset (adding an indicator for the year of survey) to allow for analysis across the years. Because the primary outcome focused on treatment for childhood diarrhea, only records that reported childhood diarrhea within the preceding two weeks were included in the study population and analyses. To determine the prevalence rate of ORS or zinc use in the treatment of childhood diarrhea, Poisson regression models were utilized. Potential confounders (including sex of child, age of child, household urbanicity, district in Bangladesh, wealth index, and education level) were identified *a priori* and adjusted for in the regression analysis. Outcome variables were treatment with either ORS or recommended home fluids (yes or no) and treatment with zinc (yes or no). To evaluate for potential disparities in zinc coverage, the study population was stratified by urbanicity (rural versus urban) and wealth quintile (poorest, poorer, middle, richer, and richest).

#### Statistical analysis.

All statistical analyses were performed using Stata software, version 17.0 (StataCorp LLC, College Station, TX) and visually represented using Microsoft Excel for Mac, version 16.61. A p-value <0·05 was considered statistically significant. Records missing data for childhood diarrhea were dropped.

### Objective #2 - other national zinc scale-up campaigns

An in-depth literature review was conducted by a research librarian in PubMed, Embase, and Web of Science using the search terms “scale-up,” “scaling-up,” “zinc,” “diarrhea,” and “diarrhoea” for articles written in English published since 2004. Only articles describing national or large-scale zinc scale-ups in countries that had Demographic and Health Surveys (DHS) data for comparison were included in the analysis. A framework synthesis was performed to systematically evaluate the various components of each zinc scale-up program using both qualitative and mixed-method evaluation. Framework analysis methodology was chosen to synthesize qualitative and quantitative research with the aim of learning about effecting change because it allowed for the combination of issues important to policy makers, practitioners, and service users, was sufficiently flexible to allow amendments to the analysis in light of the emerging literature, and led to learning specifically linked to explicit principles driving activities and their contexts [15]. A conceptual framework was constructed to accommodate the characteristics of zinc scale ups; the study designs appropriate for drawing conclusions about implementation, reach, maintenance, and effects of scaling up zinc, as well as key issues raised by policy makers, practitioners or service users or emerging from the literature during the review. Data relating to program characteristics were extracted from all included programs and organized into a table format, which was used to guide data collection and build the framework for analysis. Data were then synthesized to form meaningful statements about the zinc scale up programs. Consecutive DHS reports for each country with a zinc scale-up were obtained from 2005 through 2022. Zinc and ORS coverage rates over the years were aggregated and graphed for children under five who had diarrhea in the two weeks preceding the respective DHS Survey.

## Results

### SUZY project long-term follow-up

**Fig 1:**
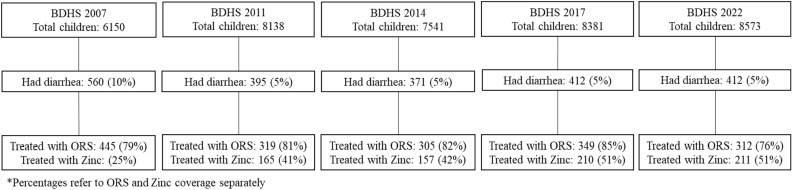
Overview of BDHS data.

Between 2007 and 2022, a total of 2150 cases of diarrheal illness in children under age five were identified in the two weeks preceding the BDHS (Fig 1). Table 1 details the demographics of those cases of childhood diarrhea. The sample populations for each of the five surveys conducted (BDHS 2007, 2011, 2014, 2017–2018, and 2022) were similar with respect to sex of child and wealth. The percentage of children aged zero to two (from 50% in 2007 to 60% in 2022, c^2^ = 42, p < 0·001) and the percentage of children from rural households (from 66% in 2007 to 75% in 2022, c^2^ = 13, p = 0·012) increased over the study period. The percentage of children receiving zinc treatment who had a caregiver that had completed secondary education or higher (from 38% in 2007 to 62% in 2017, c^2^ = 117, p < 0·001) increased from 2007–2017; however, the categories reported for education changed in 2022 and therefore were not included in this analysis.

**Table 1:** Demographics of childhood diarrhea, by DHS survey year.

	2007, n = 560	2011, n = 395	2014, n = 371	2017, n = 412	2022, n = 412	p-value
**Received Zinc**						
Yes, n(%)^*^	139 (25%)	163 (41%)	157 (42%)	210 (51%)	211 (51%)	<0·001
**Received ORT**						
Yes, n(%)	445 (79%)	319 (81%)	305 (82%)	349 (85%)	312 (76%)	0·020
**Sex of child**						
Male, n(%)	315 (56%)	217 (55%)	204 (55%)	235 (57%)	220 (53%)	0·805
Female, n(%)	245 (44%	178 (45%)	167 (45%)	177 (43%)	192 (47%)	
**Age of child (years)**						
<1, n(%)	110 (20%)	97 (25%)	85 (23%)	106 (26%)	121 (30%)	<0·001
<2, n(%)	167 (30%)	112 (28%)	115 (31%)	154 (37%)	112 (27%)	
<3, n(%)	115 (21%)	69 (17%)	62 (17%)	80 (19%)	81 (20%)	
<4, n(%)	87 (16%)	66 (17%)	60 (16%)	41 (10%)	50 (12%)	
<5, n(%)	80 (14%)	51 (13%)	48 (13%)	29 (7%)	48 (12%)	
**Urbanicity**						
Urban, n(%)	193 (34%)	108 (27%)	120 (32%)	123 (35%)	103 (25%)	0·012
Rural, n(%)	367 (66%)	287 (73%)	251 (68%)	267 (65%)	310 (75%)	
**Wealth (quintiles)**						
Poorest, n(%)	119 (21%)	99 (25%0	88 (24%)	92 (22%)	98 (24%)	0·576
Poorer, n(%)	116 (21%)	76 (19%)	82 (22%)	80 (19%)	103 (25%)	
Middle, n(%)	119 (21%)	90 (23%)	65 (18%)	91 (22%)	76 (18%)	
Richer, n(%)	97 (17%)	61 (15%)	66 (18%)	62 (15%)	58 (14%)	
Richest, n(%)	109 (19%)	69 (17%)	70 (19%)	87 (21%)	77 (19%)	
**Education** ^†^						
None, n(%)	156 (28%)	75 (19%)	57 (15%)	34 (8%)	24 (6%)	<0·001^‡^
Primary, n(%)	195 (35%)	160 (41%)	123 (33%)	121 (29%)	88 (21%)	
Secondary, n(%)	188 (34%)	132 (33%)	154 (42%)	190 (46%)	301 (73%)	
Higher, n(%)	21 (4%)	28 (7%)	37 (10%)	67 (16%)	··	

* Percentages may not add up to 100% due to rounding.

† In 2022, the DHS removed the category for higher education. We counted “Primary incomplete” and “Primary complete” as “Primary” and “Secondary incomplete” and “Secondary complete” as “Secondary”.

‡ p-value for 2007–2017.

There was a statistically significant increase in zinc use for the treatment of diarrheal illness in children under age five from 2011 to 2022 (Cochran-Armitage test: p for trend<0·001), see Table 2. There was also a statistically significant increase in use of ORS for management of diarrhea in this population between 2007 and 2017 (Cochran-Armitage test: p for trend=0·03), however there was a statistically significant decrease in the use of ORS between 2017 and 2022 (Cochran-Armitage test: p for trend=0·001). See Table 3. The percentage of childhood diarrhea cases treated with ORS or zinc from 2007 through 2022 is illustrated in S1 Fig.

**Table 2:** Adjusted zinc treatment prevalence rates and prevalence rate ratios, by year.

	2007	2011	2014	2017	2022
Prevalence Rate Ratio (95% CI)	**Reference**	1·64 (1·60–1·68)	1·68 (1·64–1·72)	2·04 (1·99–2·09)	2·04 (1·99–2·09)

**Table 3:** ORS treatment incidence rates and incidence rate ratios, by year.

	2007	2011	2014	2017	2022
Prevalence Rate Ratio (95% CI)	**Reference**	1·03 (0·75–1·40)	1·04 (0·76–1·41)	1·08 (0·79–1·46)	0·96 (0·70–1·32)

**Fig 2:**
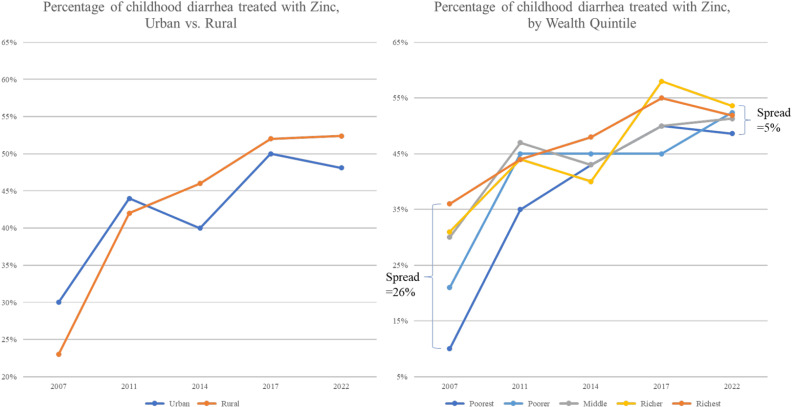
Evaluation of disparities in zinc coverage.

Zinc coverage increased in both urban and rural areas. However, the increase was greatest in rural areas; in DHS 2022, a higher percentage of childhood diarrhea cases were treated with zinc in rural areas as compared to urban areas, closing the rural/urban disparity gap. Disparities across wealth quintiles also continued to improve from DHS 2007 through DHS 2022. In 2007, the spread in zinc coverage between the richest and poorest quintiles was 26% but by 2022, all quintiles of wealth were within 5% of each other (Fig 2).

### Other national zinc scale-up campaigns

The literature review identified 12 articles describing large zinc scale-ups implemented in six other countries after the joint WHO/UNICEF zinc recommendation in 2004. While we summarize the results of our comparison here, S1 Table provides detailed descriptions of each program. Four countries (India, Kenya, Nigeria, and Uganda) ran zinc scale-up programs under the Clinton Health Access Initiative (CHAI) between 2011 and 2017 [16]. The programs in Kenya and Uganda were national scale-ups, while those in India and Nigeria worked to improve zinc coverage in three out of 28 Indian States and eight out of 36 Nigerian States, respectively [16–21]. India also had another large zinc scale-up campaign led by the Micronutrient Initiative (now Nutrition International) that ran from 2010-2015 [22]. The Point of Use Water Disinfection and Zinc Treatment (POUZN) in Nepal ran from 2005-2010 and focused on 30 out of 75 districts, representing approximately 50% of the population [23]. The Strengthening Health Outcomes through the Private Sector (SHOPS) program in Ghana promoted zinc for childhood diarrhea in three out of ten regions from 2011 to 2014 [24].

#### Funding and partnerships.

The POUZN and Micronutrient Initiative programs used program surveys to measure zinc uptake, while all others used DHS surveys. All the programs reported external funding. The Bill and Melinda Gates Foundation, UNICEF, the IKEA Foundation, and USAID were the most common funding sources. Unique to Bangladesh’s SUZY project was a partnership with a research institution, the icddr,b.

#### Program planning.

Three programs reported conducting pre-launch implementation research. Both programs in India reported formative research with consumers on ORS and zinc packaging, messaging, and knowledge gaps about zinc, however, the details are sparse about how this informed program planning. The program in Bangladesh spent nearly three years conducting operational transition to scale research studies. This included surveys of caregiver and provider diarrhea management practices, theoretical modelling of large-scale behavior change, “willingness to pay” survey for zinc treatment and the acceptability of zinc for diarrhea treatment and prevention [7]. Messaging was tailored based on interviews with caregivers that identified major motivators for treating their child with zinc. It was discovered that health professionals were concerned about the safety of zinc, especially in malnourished children, so additional safety studies were conducted and the results disseminated [25]. These methods are described elsewhere in detail [6–8,26].

#### Zinc products.

All seven scale-up programs worked to improve the quality and in-country availability of zinc products. Given the insistence of the Bangladesh Ministry of Health to scale up a locally produced product, a technology transfer was undertaken with Nutriset, the French company holding the patent for dispersible zinc tablets [8]. This resulted in a novel vanilla-flavored dispersible tablet packaged in blister packs of ten and marketed under the approved trade name Baby Zinc [25]. Nigeria, India, Kenya, Uganda, and Ghana took steps to encourage the registration of additional zinc products. The programs in Nigeria, India, and Kenya encouraged co-packs or combi-packs that included both ORS and zinc. This approach was not taken in Bangladesh because the duration of treatment is different for ORS and zinc (ORS only while symptomatic but zinc for ten days regardless of symptoms).

#### Policy changes.

The programs in Nigeria, India, Uganda, and Bangladesh added zinc to their national or state essential drug list, fostering an environment where supply chains could be strengthened and prices could be negotiated to allow for easier procurement of zinc. Scale-up programs in Nigeria, Kenya, Uganda, Bangladesh, and Ghana also worked with state and national governments to allow for over-the-counter access to zinc.

#### Training, education, and advertising.

All zinc scale-up programs included some component of training health care providers, advertising/marketing for zinc products, and educating caretakers. Since ORS coverage was already widespread in Bangladesh, the SUZY program focused primarily on promoting zinc, while all other programs promoted both ORS and zinc, either through co-packs (Nigeria, India, and Kenya) or as separate components (Uganda, Ghana, and Nepal). Training for health care providers was similar across the programs, with the exception of Bangladesh which included educating ‘village doctors’ (unlicensed health workers) who were the first source of care for more than 70% of childhood diarrhea cases. Most of the zinc scale-up programs used radio, TV, and mass media to market ORS and zinc products, and nearly all provided some form of education for caretakers through mass media messages. The zinc scale-up program in Bangladesh engaged a marketing firm, Dhansiri, that guided a mass media campaign involving newspapers, TV, and radio ads informed by a social marketing framework. Key messages and motivators used in the campaign were identified through pre-launch implementation research. They also used newspapers, billboards, a TV drama series, sponsored cultural events, and a Baby Zinc logo theme song to promote zinc uptake. In addition, the program in Nigeria used “diarrhea champions” to promote zinc through schools and churches, Kenya’s scale-up established ORS corners (on site rehydration for sick children), and Uganda implemented Behavior Change Communication activities to increase patient demand for ORS and zinc.

#### Knowledge transfer and dissemination.

All of the programs published their methods and results in peer reviewed research journals. We were not able to capture and compare other methods of dissemination (such as conference presentations, workshops, and donor-sponsored dissemination events) for programs besides Bangladesh. The scale-up program in Bangladesh actively promoted knowledge transfer through several avenues, with the goal of increasing zinc coverage not just in Bangladesh but throughout the world. These included a project website, a biannual newsletter in both Bangla and English that reached ~20k health workers, and an annual international zinc conference [25].

#### Comparison of results.

**Fig 3.**
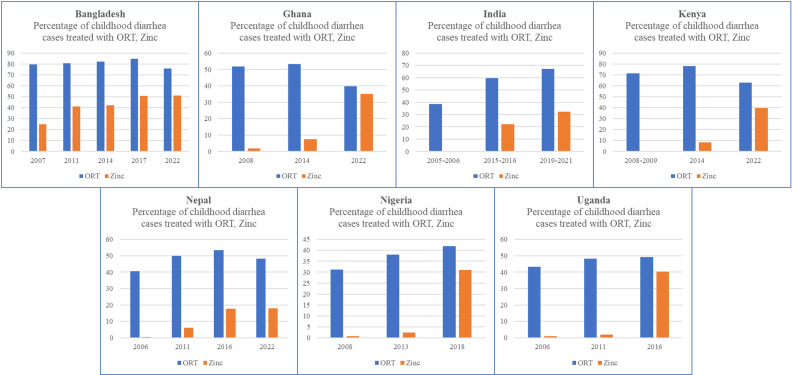
ORS and zinc coverage, by country, DHS data only.

Fig 3 shows the percentage of childhood diarrhea cases treated with ORS or zinc in each of the seven countries that implemented large-scale zinc programs. These countries all started in different places with regards to their zinc and ORS coverage for childhood diarrhea. Four of the seven (Bangladesh, Ghana, Kenya, and Nepal) had ORS coverage at or >50% prior to their scale up programs. India had the largest increase in ORS coverage, rising from 38·5% in 2005 to 57·3% in 2021. Bangladesh started (79·5% in 2007) and ended (75·7% in 2022) with the highest rate of ORS coverage, despite a decrease in ORS coverage during the COVID-19 pandemic (2017 coverage was 85·2%). All the countries started with zinc coverage rates <10%. Kenya saw the largest increase in zinc coverage, rising from 0·2% coverage in 2008 to 39·5% coverage in 2022. Bangladesh started and ended with the highest zinc coverage levels.

**Fig 4:**
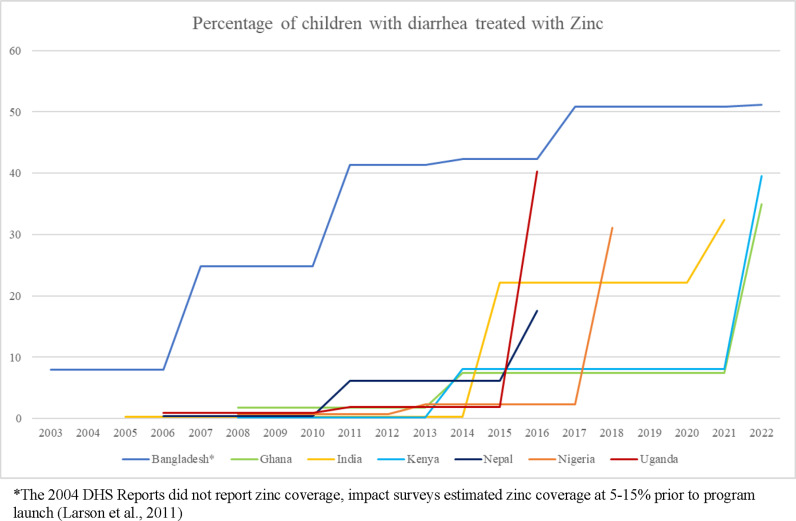
Zinc coverage, includes approximate program dates and zinc data prior to 2005.

Fig 4 shows the percentage of children under age five with diarrheal illness treated with zinc over time by country. Despite having similar starting points with regards to zinc coverage, the effectiveness of the zinc scale-up programs varied widely. While several countries had a large increase in zinc coverage between 2017 and 2022, there is a large spread between the country with the highest rate of zinc coverage (Bangladesh) and the country with the lowest rate of zinc coverage (Nepal) in the most recent DHS report.

## Discussion

As the first national level zinc scale-up program, Bangladesh has experienced sustained and consistent uptake of zinc as a treatment for childhood diarrhea. Since the conclusion of the SUZY program in 2008, zinc coverage has continued to increase and disparities in zinc coverage identified in the short-term follow-up (previously favored wealthy, urban households) resolved on long-term monitoring.

Not only has the prevalence of zinc coverage increased, but the BDHS data show an overall decrease in the prevalence of diarrhea in Bangladesh; the DHS surveys have reported a decrease in diarrheal prevalence from 10·8% in 2007 to 4·8% in 2022 [10–14]. Deaths in children under 5 years of age due to diarrheal illness have also decreased. In 2003, diarrheal disease was the seventh leading cause of death in Bangladesh, with a death rate of 66·5 per 100,000 children [1]. The most recent data from 2019 show that deaths from diarrhea have dropped out of the top ten and are now ranked 11th with a death rate of 12·62 per 100,000 children [1].

Since the WHO/UNICEF revised their clinical management of childhood diarrhea guidelines to include zinc in 2004, seven countries have published studies addressing the initiation of large-scale programs to implement this lifesaving measure. Each zinc scale-up program was effective but to varying degrees. Nepal’s POUZN program reported the lowest increase in zinc coverage (0·4% in 2006 to 17·9% in 2022), while Bangladesh’s SUZY program resulted in a substantial increase (8% in 2006 to 51·2% in 2022). The top three countries for zinc coverage rates (Bangladesh, Kenya, and Uganda) implemented nation-wide scale-up programs, rather than focusing on specific regions.

The COVID-19 pandemic strained health systems and disrupted supply chains globally, with many health services having not returned to pre-pandemic levels [27]. The drop in ORS coverage in Bangladesh from 85·2% in 2017 to 75·7% in 2022 can likely be attributed to pandemic-related workforce issues and limited access to supplies. Ghana and Kenya also reported decreases in ORS coverage in 2022; however, this could be attributed to the fact that their indicators changed slightly (Ghana reported ORS only and Kenya reported “ORS or increased fluids” rather than the “ORS or recommended home fluids” indicator that had been used previously), so the data may not be directly comparable to previous years. Despite the pandemic, all countries with 2022 DHS data (Bangladesh, Ghana, India, Kenya, and Nepal) reported an increase in zinc coverage between 2014 and 2022.

This report is the first of its kind in that it takes a global look at all zinc scale-up programs reported in the literature to date. Globally, over 500,000 children die each year from diarrheal illness, and these deaths are largely preventable. The importance of scaling up programs for inexpensive and effective treatments such as zinc cannot be overstated. Having a comprehensive understanding of the various components of the zinc scale-up programs is essential to designing future scale up programs that are effective. The countries that had the highest zinc coverage rates (Bangladesh, Kenya, and Uganda) as well as the largest increase in zinc coverage (Kenya and Uganda), all had national scale-up programs.

In addition to being a national zinc scale-up program, the SUZY program had several other factors that set it apart from the other zinc programs. First, the SUZY program focused solely on increasing zinc coverage. ORS was already well established in Bangladesh at the beginning of the SUZY program so program planners could focus all their efforts on promoting zinc. As other programs worked on simultaneously increasing ORS and zinc coverage, targeted messaging for these countries was not possible since ORS and zinc treatments vary in duration and have different mechanisms of action. Second, by selecting a single form of zinc to promote for diarrheal treatment, the SUZY authors ensured that Baby Zinc was uniquely positioned as a treatment for childhood diarrhea since zinc syrups were already available and promoted for a wide range of ailments. The other programs promoted multiple zinc products, making it less of a targeted treatment for childhood diarrhea. Third, the advertising, marketing, and educational plan for the SUZY project went beyond the promotion campaigns of the other scale up projects, creating a TV drama series, cartoons, and a memorable jingle to help the message stick. And finally, the unique partnership between the SUZY project and their research partner, icddr,b, allowed for more pre-implementation research that guided the roll-out of the zinc scale-up. This social marketing campaign aimed to reach rural and urban poor households in order to reduce disparities in zinc usage. Extensive research conducted over the three-year period made sure that Baby Zinc was uniquely positioned as a treatment, helped to set a target price range that people would be willing to pay, addressed specific provider concerns about zinc, and targeted caregiver messages to their priorities. These factors likely contributed to the closure of the urban/rural disparity gap and the reduction in wealth disparity that we observed in this study.

Our study did have several limitations. First, the DHS does not include data on whether treatment was completed, so it was not possible to evaluate the proportion of children who completed the treatment course. At the time of this analysis, Uganda had not had a DHS report published since its program concluded; therefore, the full impact of its program may not be reflected in the DHS report data. The most recent DHS survey for Bangladesh (2022) was only available in the form of a preliminary report at the time of this analysis – more detailed data will be available when the final report is published. For some countries (Ghana and Kenya), the indicators used to report ORS were changed in 2022, making it difficult to compare ORS coverage across time. Two of this paper’s authors (TK and CL) participated in the SUZY scale up campaign thus providing detailed information not available from the other reported programs. Future research should include in-depth analysis using full DHS data for other LMICs in order to compare in detail the long-term effectiveness of zinc scale-up programs in different countries.

## Conclusions

The SUZY program was the earliest and most comprehensive zinc scale-up program with regards to pre-launch implementation research and scope of marketing preparation and conduct. The extensive implementation research that was conducted likely contributed to the success of the scale-up launch of zinc, as did the marketing program that was simultaneously broad in scope and at the same time, targeted to caregivers’ priorities and concerns. This success has been demonstrated, both in the short term and now, over a decade after the project concluded (Objective #1). The methods from the SUZY project have stood the test of time, making a lasting impact on childhood diarrhea treatment in Bangladesh and saving thousands of lives. Objective #2 shows that some of the most successful zinc scaling up programs have been national level scaleups with significant pre-launch implementation research addressing key identified knowledge gaps and partnering with research organizations to assist. Multidisciplinary teams and creative and memorable marketing strategies prioritizing caregiver-specific messages are also important programmatic elements to include when designing and implementing effective zinc scaling up programs.

Zinc saves lives. By knowing the elements that create a successful zinc scale-up with lasting impacts, diarrheal disease deaths can be reduced globally and 500,000 children annually can have a chance at life.

## Supporting information

S1 Fig Percentage of childhood diarrhea cases treated with ORS, Zinc. (TIF)

S2 Fig Zinc Coverage, includes approximate program dates and zinc data prior to 2005, black and white. (TIF)

S1 Table Framework synthesis of zinc scale-up programs. (XLSX)
