# Supplementary figures and images for: Sustainability of zinc coverage for acute childhood diarrhea in Bangladesh and other low- and middle-income countries: one decade following the SUZY project
Source: PLOS Glob Public Health. 2025 Feb 10;5(2):e0004265. doi: 10.1371/journal.pgph.0004265 (PMC11809797; doi:10.1371/journal.pgph.0004265)

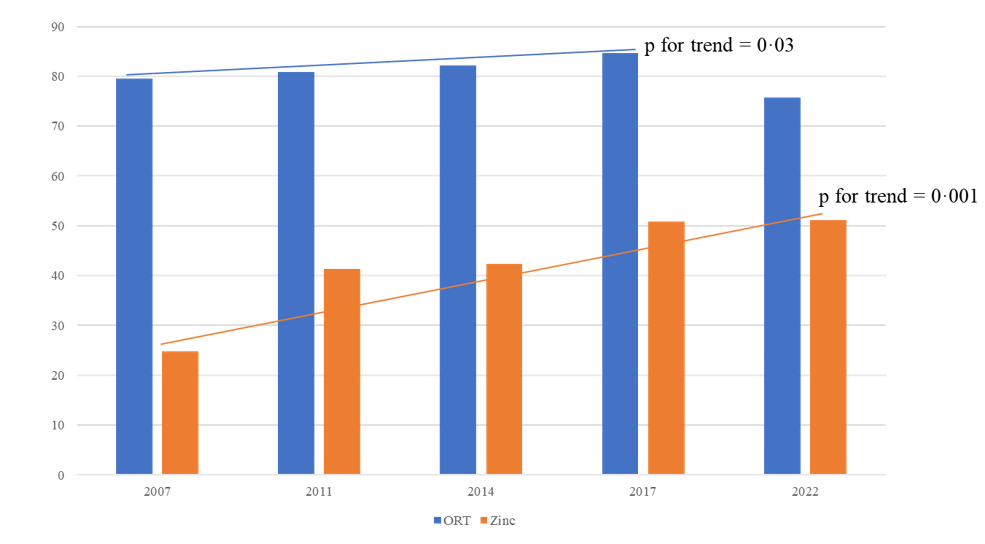

Supplement: S1 Fig — (TIF) [file pgph.0004265.s001.tiff]

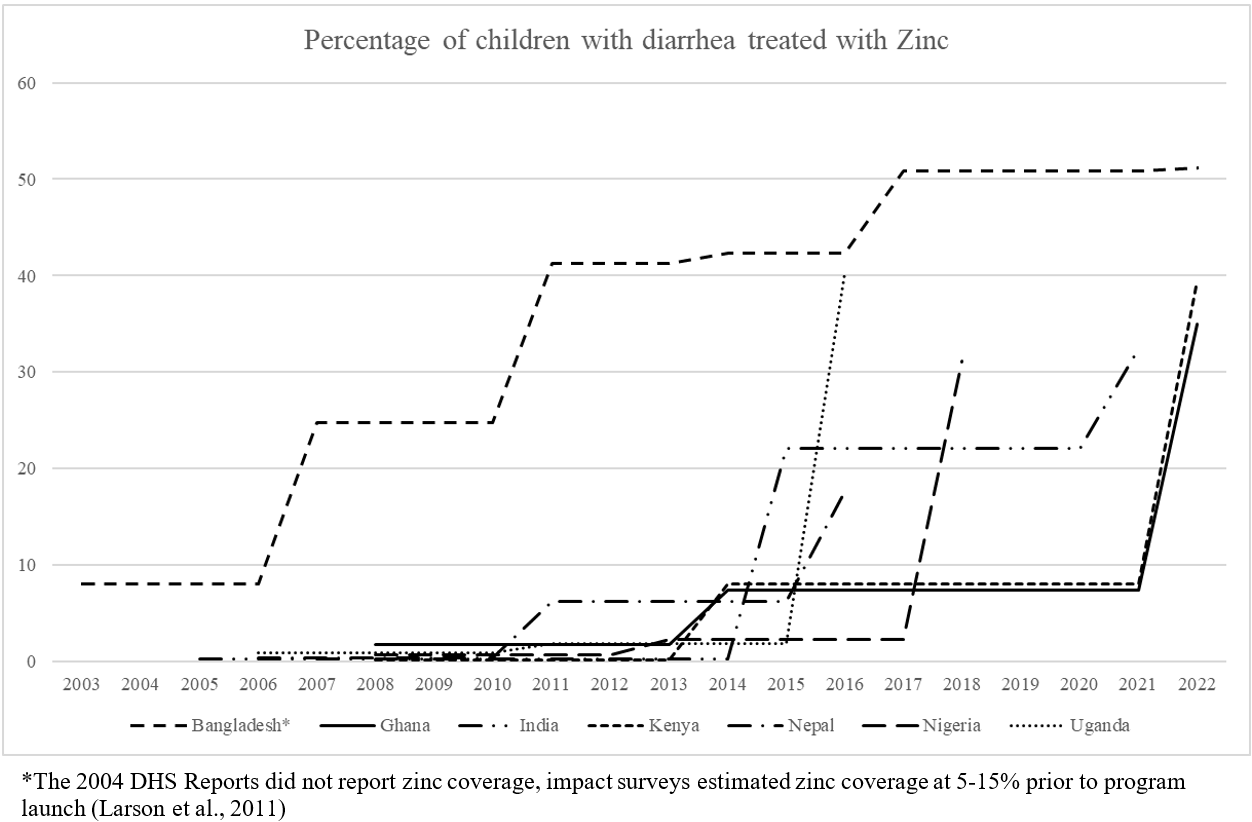

Supplement: S2 Fig — (TIF) [file pgph.0004265.s002.tif]
